# Resolidification of a mushy-zone and directional solidification: a method for efficient alloy development demonstrated using the example of Cu–Ga–Sn

**DOI:** 10.1038/s41598-020-78772-7

**Published:** 2020-12-10

**Authors:** Martin Salge, Gunther Wiehl, Klaus Hack, Markus Rettenmayr

**Affiliations:** 1 Friedrich-Schiller-Universität Jena (Germany), Otto Schott Institute of Materials Research, Löbdergraben 32, 07743 Jena, Germany; 2 Formerly Saxonia Technical Materials, Rodenbacher Chaussee 4, 63457 Hanau, Germany; 3 GTT Technologies, Kaiserstraße 103, 52134 Herzogenrath, Germany; 4 ifw Jena, Ernst-Ruska-Ring 3, 07745 Jena, Germany

**Keywords:** Materials science, Metals and alloys

## Abstract

An experimental method for alloy development that allows to systematically scan multicomponent alloy systems is presented using the Cu–Ga–Sn system as an example. Rods with homogeneous concentration distribution of different initial compositions are annealed in a steep temperature gradient with temperatures in the range from above liquidus to below solidus temperature. During resolidification of the initially formed mushy-zone, a continuously varying composition over the length of the rods develops. Further concentration gradients of the alloying elements are generated during subsequent directional solidification. The graded samples are evaluated for different properties. Vickers hardness as a function of composition was measured along the length of the samples to get first information on the mechanical behavior of bulk samples. The melting range of selected compositions (cylindrical disks of 1 mm thickness cut out of the rods) was determined by differential scanning calorimetry and compared to liquidus temperatures extrapolated from the binary systems with a fitting method and the Calphad method. With the procedure introduced here, it is possible to determine several alloy properties over an extended composition range of a multicomponent system with significantly reduced experimental effort.

## Introduction

Considering the increasing complexity of technical materials and the recent development strategy of alloys containing five or more principal elements^1–3^, classical alloy development strategies, e.g. build up data bases starting from binary alloys, tend to be tedious. The determination of phase equilibria of highly multicomponent systems is unlike to be carried out for a large number of alloys systems: the experimental effort to investigate alloy systems for targeted properties is rapidly rising with each additional constituent element. Techniques for high throughput experimentation have been presented for fast synthesis of a multitude of alloy compositions. While functional properties are then generally accessible to measurement, mechanical properties of bulk material such as hardness or strength cannot be characterized without considerable further effort. A majority of today’s high throughput techniques used for alloy development focuses on preparing and investigating thin films^4^, even though it is known that the properties of bulk material and thin films can differ significantly^5–7^. A bulk high throughput preparation technique would offer the advantage to get realistic information about phase formation and mechanical properties of complex systems and their sub-systems. A few publications on bulk samples with a concentration gradient have been presented in the literature, linking compositional variations to property changes like lattice parameter^8^, phase fractions^9^ and mechanical properties^10^. In recent years, different bulk high throughput methods for investigating the complex material class of high entropy alloys (HEAs) have been developed for accelerated preparation and processing of compositionally graded alloy samples. These include combinatorial laser additive manufacturing (LAM) techniques^11–13^, diffusion couples/multiples^14–16^ and rapid alloy prototyping^17^.

In this work, mushy-zone resolidification in a temperature gradient is used for preparing sample rods with longitudinal concentration gradients of the alloying elements. This technique has already been utilized to determine solid/liquid equilibria^18^, solvus composition paths in multicomponent alloys^19^ as well as frequency factor and activation energy of diffusion coefficients in solids^20^, efficiently reducing the experimental effort compared to conventional strategies.

For the present work, the Cu–Ga–Sn system was chosen as a model system. The binary alloy sub-systems are well known^21–25^, whereas the ternary phase diagram has not been established reliably yet. Both Sn and Ga exhibit considerable solubility in Cu; solidus and solvus lines can thus be determined accurately, even if the concentration distribution is not entirely homogeneous. Cu–Ga–Sn alloys have potential applications as shape memory alloys^26^ or special bronze alloys with properties deviating from the classical Cu–Sn bronze due to a higher degree of freedom in designing microstructure and functional properties.

The aim of the present paper is to introduce a method that evaluates concentration gradients, allowing the mapping of bulk material properties and the measurement of solidus/liquidus temperatures as a variety of a high throughput technique. Additionally, different approximative methods to predict liquidus temperatures in ternary systems are tested and compared.

## Results and discussion

When the upper end of an alloy rod is melted in a temperature gradient, a mushy-zone with varying solid and liquid phase fractions will form between the initial liquidus temperature (liquid fraction f_L_ = 1) and solidus temperature (f_L_ = 0). At each temperature throughout the gradient, the concentrations of the solute elements in the solid and liquid phases will rapidly adjust to the local equilibrium concentrations as given by the phase diagram (see Method section, Fig. 7). The gradient in phase fraction thus transforms to a concentration gradient in liquid and solid. This in turn results in mass transport of solute elements towards higher temperatures, i. e. lower concentrations in the case of alloys with a melting point depression (partition coefficient of k < 1). The mass transport out of the mushy-zone occurs mostly through the liquid phase and reduces the solute concentration within the mushy-zone. Reducing the concentration of the melt that is at its equilibrium concentration leads to change of phase fraction, i.e. solidification with the equilibrium concentration corresponding to each location. The mushy-zone gradually resolidifies from lower to higher temperatures, while the concentration gradient along the sample length in the former mushy-zone is maintained. During subsequent directional solidification, a further gradient can form, if steady-state solidification is avoided. This can e.g. be achieved by forced convection due to electromagnetic stirring.

**Figure 1:**
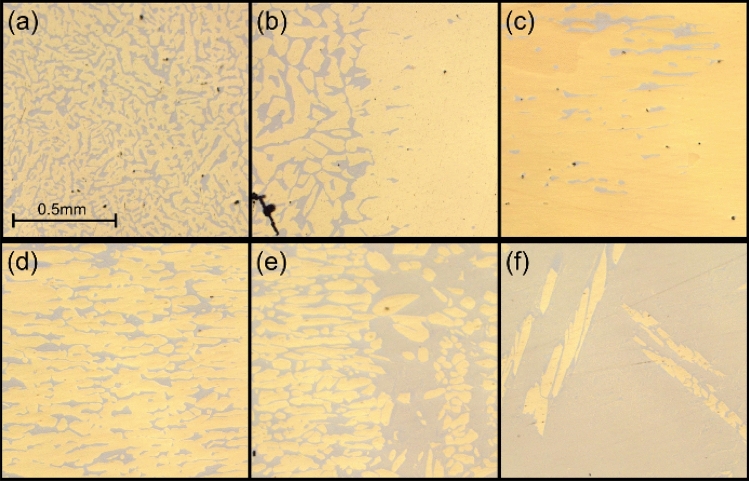
Optical micrographs taken along the resolidified Cu_86_Ga_5_Sn_9_ sample at positions with increasing temperature: (**a**) as-cast, (**b**) microstructural transition initial solidus temperature, (**c**) mostly α-phase and a small fraction of δ, (**d**) increasing fraction of δ, (**e**) high δ fraction and (**f**) α in δ-phase.

Figure 1 shows typical microstructures along the former temperature gradient after mushy-zone resolidification and subsequent directional solidification of a Cu_86_Ga_5_Sn_9_ (all concentrations in at.-%) alloy rod. At the lower (cold) end of the sample, the as-cast microstructure (a) consists of dendritic primary α-phase (yellow) and interdendritic Sn-rich δ-phase (grey); with increasing temperature, the microstructure shows an increase in the phase fraction of α and gets coarser (b); at the location of the initial solidus temperature, the δ-phase disappears; towards higher temperatures, the δ-phase reappears (c) with increasing phase fraction, resulting in a structure of elongated α-phase regions surrounded by δ-phase (d); a change to a higher fraction of δ-phase occurs close to the initial liquidus temperature (e). Towards further increasing temperatures, the fraction of α-phase decreases further until the microstructure consists mostly of δ-phase (f).

**Figure 2:**
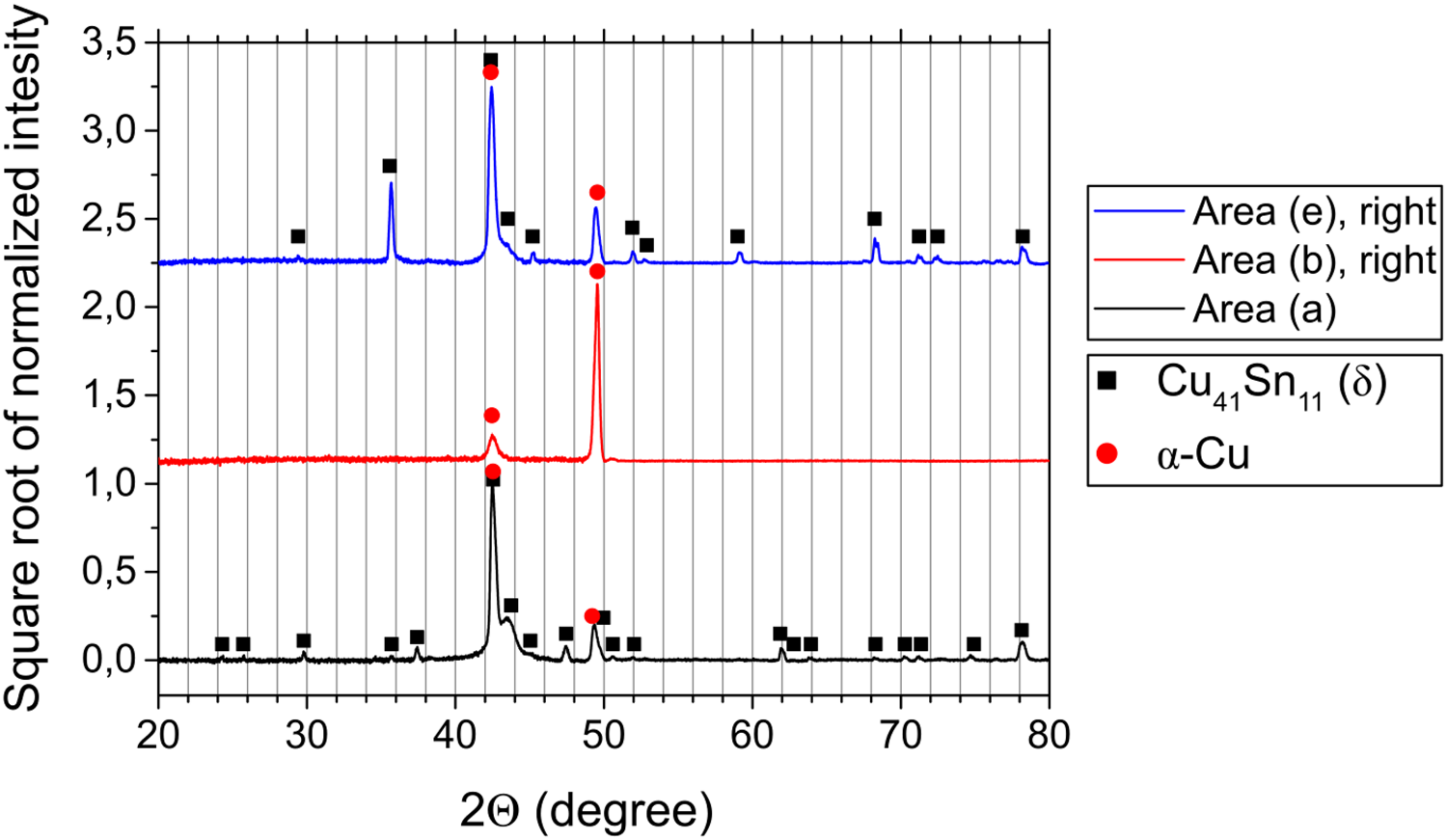
Diffraction patterns of Cu_86_Ga_5_Sn_9_ measured at three different positions along the sample rod, with the designation of the measured areas referring to Fig. 1. For a clearer view, the intensity range of areas (**b**) and (**e**) was increased.

In order to confirm the phases as α and δ, X-Ray Diffraction (XRD) measurements were carried out at several locations of the Cu_86_Ga_5_Sn_9_ bulk sample rod. Diffraction patterns of three different areas along the sample are shown in Fig. 2. The pattern of the as-cast microstructure (area (a)) confirms that the two phases are indeed α and δ. The single-phase region following the as-cast microstructure does not show any characteristics of the δ phase, but two characteristic peaks of α-Cu. The diffraction pattern of the right side of area (e) looks very similar to the pattern of the as-cast microstructure. At the locations with the second phase, characteristic peaks of the δ-phase are reappearing, thus leading to the conclusion that the two phases are again α and δ. Additionally, the compositions of both phases were measured at several locations along the sample using Energy Dispersive X-ray (EDX) analysis. An average composition of Cu_87.4_Ga_7.7_Sn_4.9_ was obtained for the α-phase, and Cu_78.7_Ga_2.8_Sn_18.5_ for the δ-phase, both exhibiting only slight variations along the sample length. This implies that the change in average composition along the sample length is due to the change in phase fractions.

It is worth noting that in comparison with XRD patterns of powder samples or very fine grained samples, the reflections in Fig. 2 are of a different intensity, and some reflections are not detected at all. This is attributed to the fact that the coarse grained sample only offers a few orientations to be analyzed rather than the full X-ray ”cone” as in powder diffraction. Additionally, preferred orientation of crystallites (texture) may further enhance this effect. Nonetheless, the diffractograms in Fig. 2 indicate that it is possible to identify the phases in the microstructures without the need to generate powders for the XRD analysis.

**Figure 3:**
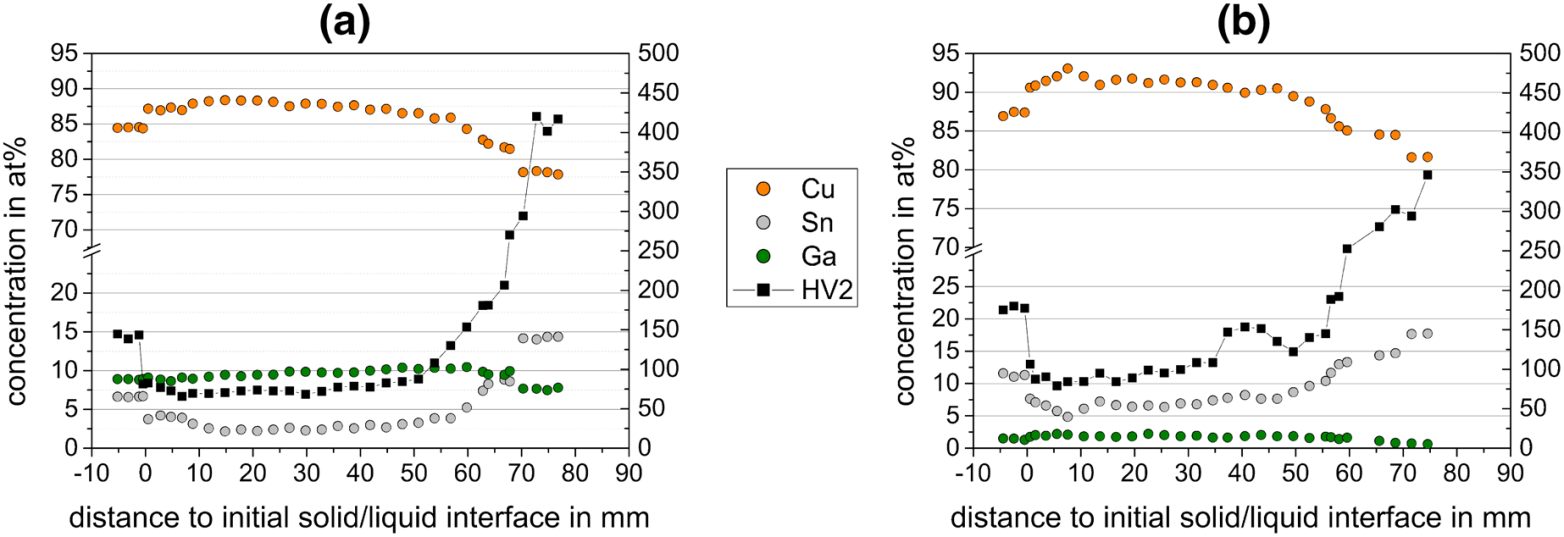
Composition and Vickers hardness HV2 change at positions relative to the initial solid/liquid interface of (**a**) Cu_84.5_Ga_9.5_Sn_6_ and (**b**) Cu_87_Ga_2_Sn_11_.

EDX measurements were performed to correlate the microstructures with the change of average composition along the length of the sample rods. The size of measurement window was 1 × 3 mm^2^, and a distance of 1 mm between each measurement window was chosen for a combination of speed and resolution. Particularly, jumps in composition at transition temperatures were to be resolved. The correlation of compositional change and Vickers hardness (HV2) are shown in Fig. 3 for samples with the composition Cu_84.5_Ga_9.5_Sn_6_ and Cu_87_Ga_2_Sn_11_. The course of the concentration profiles and the changes in microstructure along the rod length were, in principle, the same for all samples. All samples exhibit compositions of the as-cast microstructure at the lower (cold) end that agree well with the targeted compositions (Table 1). At the position of the initial solidus temperature, a sharp decrease in Sn content and simultaneously an increase in Cu content occur, while the amount of Ga remains essentially constant. This sudden change in composition is followed by a linear decrease of the Sn and increase of the Cu concentration. At the solidus temperature, the abrupt decrease in Sn content causes a transition from the two-phase as-cast microstructure (Fig. 1a) to a single-phase structure (Fig. 1b) by partial melting and resolidification of the Sn-depleted Cu solid solution. Subsequentially, the Sn and Cu concentrations exhibit Scheil-type profiles (i.e. an initially flat and then continually increasing slope of the profiles), whereas the Ga content varies only slightly. The continuous enrichment of Sn in the melt causes the reappearance of the δ phase and then an increasing phase fraction (Fig. 1c,d). The second abrupt transition in the microstructure (Fig. 1e) is correlated to a further sudden increase in Sn-concentration. This can be correlated with the peritectic reaction, which is also accompanied by a sudden change of the phase fractions. Above this microstructural transition, the microstructure consists of a δ-matrix with embedded α phase (Fig. 1f). The jump in concentration occurring at ~ 70 mm above the initial solid/liquid interface (see Fig. 3a) is due to quenching at the end of the experiment.

**Table 1:** Targeted compositions of the four sample rods compared with results of EDX measurements in the as-cast microstructure.

Sample	Targeted comp. (at%)			Measured comp. (at%)		
Number	Cu	Ga	Sn	Cu	Ga	Sn
1	87.0	2.0	11.0	87.3	1.4	11.3
2	86.0	5.0	9.0	85.6	4.4	10.0
3	84.5	9.5	6.0	84.5	8.9	6.6
4	83.0	14.0	3.0	83.1	13.7	3.2

As expected, the Vickers hardness along the sample rods approximately correlates with the solute concentration: a constant hardness in the as-cast microstructure, a hardness decrease in the resolidified mushy-zone followed by a hardness increase at the upper end similar to the Scheil-type concentration increase. Due to the solute enrichment in the liquid phase during the directional solidification, the concentration of Sn in the quenched liquid is significantly higher than the initial concentration in the as-cast structure, and so are the measured hardness values.

**Figure 4:**
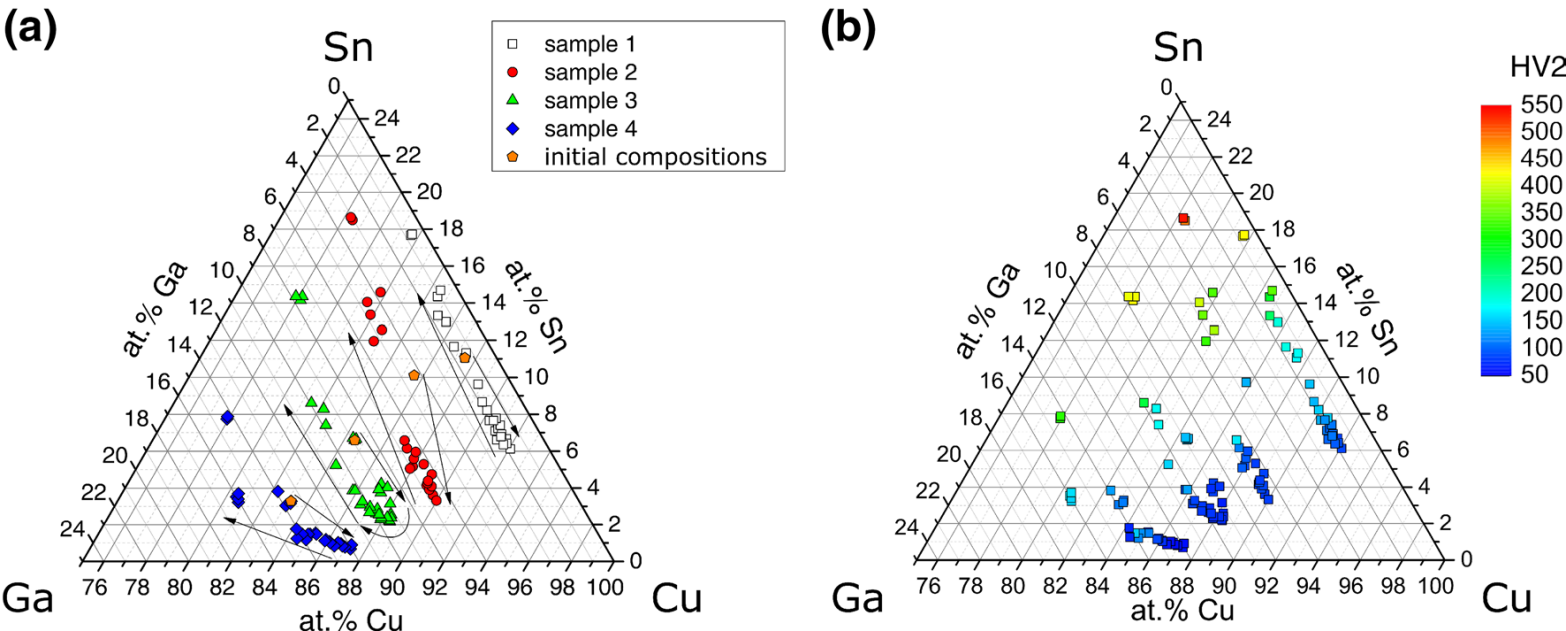
Cu-rich corner of the Cu–Ga–Sn system with (**a**) solidification paths of the gradient samples with arrows indicating the direction of change, and (**b**) Vickers hardness (HV2).

The Cu-rich corner of the Cu–Ga–Sn ternary system containing the solidification paths of four sample rods is shown in Fig. 4a. Starting with a Cu content of ≥ 82 at%, each sample shows an increase in its Cu-concentration at positions above the initial solidus temperature, reaching a maximum between 88 and 92 at%, followed by a steady decrease to ≤ 78 at% Cu. The path of the sample with the highest Sn content can be represented by a straight line (almost isopleth) with Ga contents of 1…2 at%. With higher Ga content, the variation of Ga concentration along the solidification path increases, leading to more curved paths for the samples with 5…14 at% Ga.

The path of the Vickers hardnesses (HV2) along the samples is shown for all samples in Fig. 4b. The hardness increases with decreasing Cu concentration; Sn-rich alloys exhibit higher HV2 values than Ga-rich ones. In general, the measured hardness values are in good agreement with literature values. For pure Cu, a hardness of around 35HV is given, while Cu–Sn alloys exhibit hardness values of more than 140HV, increasing with the Sn content^32,33^.

**Figure 5:**
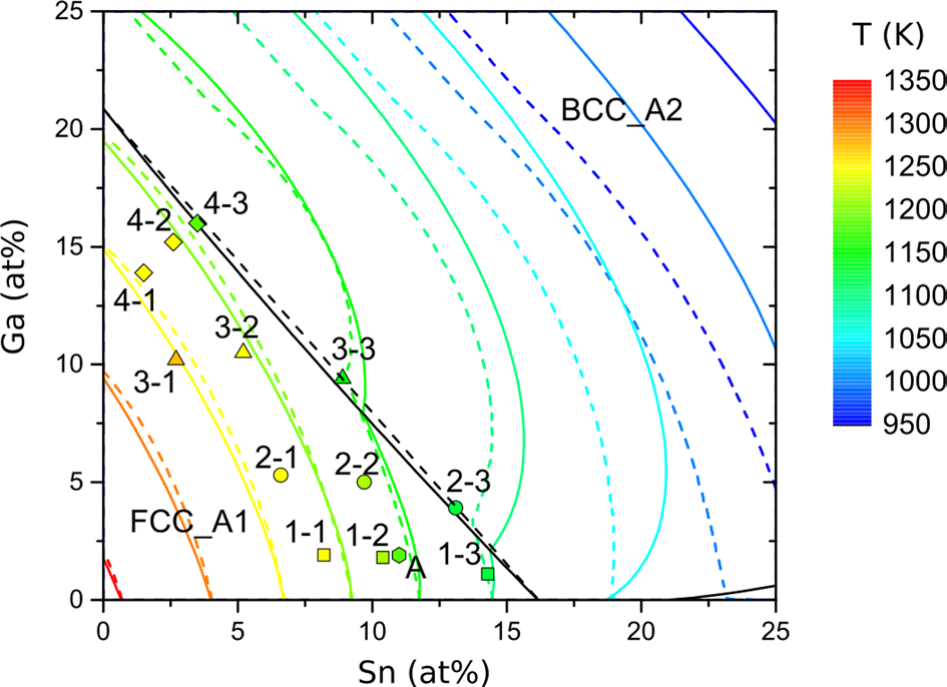
Liquidus projection and compositions of three samples cut from each of the sample rods for DSC measurements (1–1 to 4–3) and a bulk Cu_87_Ga_2_Sn_11_ sample (A) with the color filling showing the measured liquidus temperature; the dashed liquidus lines were obtained using polynomial extrapolation from the binary systems, the solid lines were calculated with FactSage; black lines separate regions where the liquid is in equilibrium with different phases (FCC_A1 and BCC_A2, respectively), i.e. they represent univariant equilibria; the solid black line was calculated with FactSage, the dashed line is an approximation by connecting the peritectic concentrations of the liquids in the binary systems.

For measuring the melting range for different alloy compositions, three slices of 1 mm thickness were cut from each rod at particular regions of interest. For example, slice 1–1 was cut out right after the original solid/liquid interface, 1–2 just before the transition from a low to a high fraction of δ-Phase, and 1–3 right after the transition. The sample slices 2–1 to 4–3 were cut out at equivalent positions from the other rods. DSC measurements up to 1000 °C were performed with a heating/cooling rate of 10 K/min. The onset of the first DSC signal and peak temperatures of the last DSC signal were assigned to the solidus and liquidus temperatures, respectively (Table 3). Figure 5 shows the compositions of the DSC samples and two types of liquidus lines, solid and dashed. The solid lines were extrapolated using the Calphad method, particularly the thermochemical software FactSage^34^ together with a database set up for the ternary system Cu–Ga–Sn using the data for the three constituent binaries. The dashed lines were extrapolated from the binary systems “geometrically” by adding up the contributions to the melting point depression of the two alloying elements in a non-linear multi-binary approach.

For this, the calculated liquidus lines of the binary sub-systems Cu–Ga^24^ and Cu–Sn^35^ were taken from the literature and fitted using polynomial functions of the form

1 $${T}_{L,i}= {T}_{m}+ {A}_{i} {c}_{i}+{B}_{i}{c}_{i}^{2}+{C}_{i}{c}_{i}^{3}$$

with the melting point of pure Cu T_m_, the concentration of the i-th (i = Sn, Ga) alloying element c_i_ and fitting parameters A_i_, B_i_ and C_i_. The values of the coefficients for composition ranges in the binary systems with different curvatures are shown in Table 2. After determining the fitting parameters, the ternary liquidus temperature can be calculated as

**Table 2:** Coefficients for the polynomial fitting functions of the liquidus lines for the binary systems Cu–Sn and Cu–Ga. For Sn and Ga concentrations > 16.3 at% and > 21.0 at%, parabolic fitting functions were used. As a measure for the fit quality, adjusted R^2^ values are given for each set of parameters.

Concentration range	T_m_ (K)	A	B	C	Adj. R^2^
Sn ≤ 16.3 at%	1358.2	− 11.04952	− 0.97739	0.03748	0.99969
Sn > 16.3 at%	1051.6	9.46055	− 0.50151	0	0.99909
Ga ≤ 21.0 at%	1358.2	− 3.20656	− 0.32792	0.00431	0.99998
Ga > 21.0 at%	1252.5	− 0.27239	− 0.13735	0	0.99992

2 $${T}_{L}= {T}_{m}+ \sum_{i}{A}_{i} {c}_{i}+{B}_{i}{c}_{i}^{2}+{C}_{i}{c}_{i}^{3}$$

**Figure 6:**
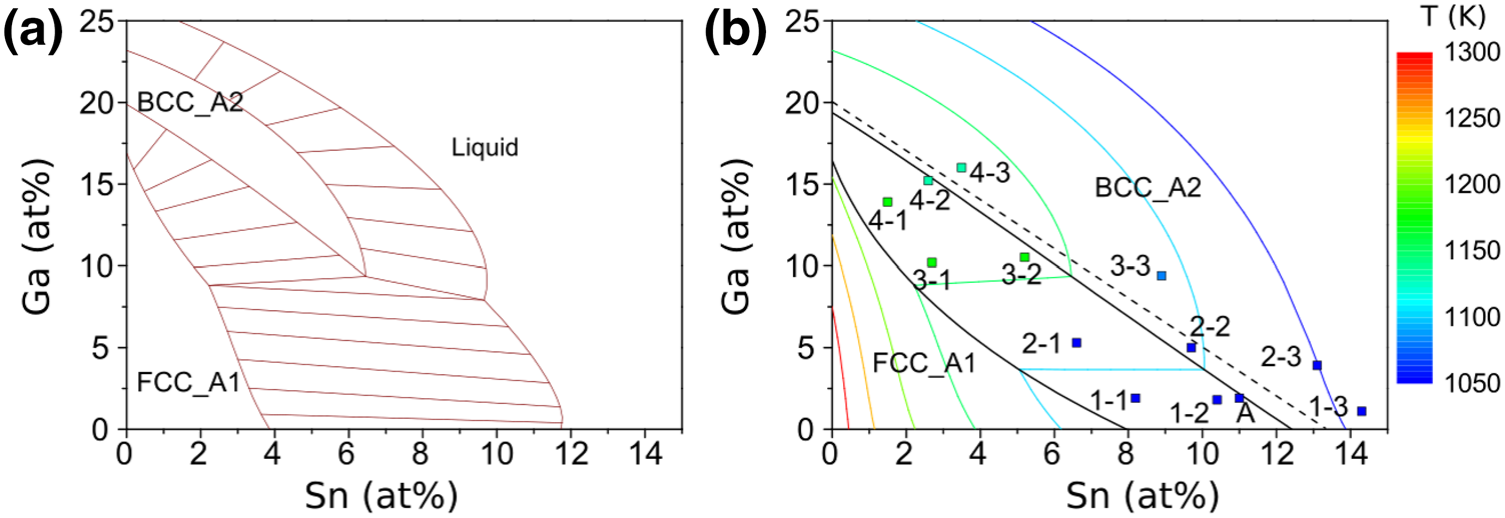
(**a**) Isothermal section at 1150 K, and (**b**) solidus projection calculated with FactSage^34^. Compositions of the DSC samples (1–1 to 4–3) and a bulk Cu-2 at% Ga-11 at% Sn sample (A) are shown with the color filling indicating the measured solidus temperature. Black lines represent univariant equilibria; the dashed line was obtained considering the microstructural transitions from α to δ as shown in Fig. 1e.

In both extrapolation methods, the solute elements were treated independently, ternary interactions are not known and not considered. Similarly, as in the binary sub-systems Cu–Sn and Cu–Ga, the ternary system is expected to exhibit a transition from an equilibrium of liquid and the α-FCC-phase (“FCC region”) to an equilibrium of liquid and β-BCC-phase (“BCC region”) in the Cu-rich corner. As a simple approximation for the Sn and Ga concentrations at which the transition occurs, the transition points from the binary systems at 21.0 at% Ga and 16.3 at% Sn were connected by a straight line (Fig. 5, black dashed line). Inside the resulting triangular FCC region, the liquid is assumed to be in equilibrium with the FCC phase and Eq. 2 was used to calculate the liquidus isotherms. For compositions outside this range, the liquidus temperatures were calculated iteratively for a constant Ga concentration using the derivative of the polynomial fitting function for Sn > 16.3 at%. Since Ga shifts the transition from the FCC to BCC region towards lower Sn concentrations, the Sn concentration for the calculation of the derivative value has to be modified by a term depending on the Ga concentration. The modification term can be derived from the transition between the FCC and BCC region, leading to the modified concentration c’_Sn_ = c_Sn_ + c_Ga_/1.2883, 1.2883 being the slope of the boundary line (black dashed line in Fig. 6). A step width of 0.04 at% for the Sn and Ga concentrations was used in this calculation.

The liquidus isotherms generated by FactSage were calculated based on the available information concerning phase stability and interactions in the binary systems. In the FactSage extrapolation, the ternary interactions are also not considered, whereas the binary interaction terms of Cu-Sn and Cu-Ga are adequately accounted for, and the binary interaction term of Sn-Ga is estimated to be positive, i.e. repulsive (6.7 kJ/mol). The α-FCC_A1 and the β-BCC_A2-phases exhibit stability ranges in both Cu–Sn and Cu–Ga and are taken to be the same phase in the ternary composition range. The γ-DO3-phase in Cu-Sn and the γ-D83-phase in Cu-Ga were regarded as two separate phases. Seeing that in the experimental microstructure only 2 phases appear, this is expected to be of minor importance for a comparison of measured and extrapolated values.

A comparison of the lines calculated using the Calphad method with the geometrically extrapolated ones shows similar liquidus isotherms in the FCC region with only minor deviations. On the binary Cu–Sn edge, the liquidus temperatures obtained by the extrapolation are the same as the ones calculated with FactSage, while on the Cu–Ga edge a small difference of 0.1…0.3 at% is noticeable. The FactSage prediction of the boundary between the FCC and BCC regions is not a straight line as assumed for the geometrical extrapolation, but the deviation from linearity is quite small. For the FCC region, the liquidus lines of both approaches agree well. At the transition to the BCC region, the curvature of both types of lines changes drastically, with increasing deviation between them. While the liquidus lines for 1150 K and 1100 K are still quite close to each other with a similar curvature, geometrically extrapolated lines for lower temperatures are much closer to each other than the calculated ones and exhibit a different curvature. In general, the ”geometrical” model predicts lower liquidus temperatures than the FactSage calculation for compositions in the BCC region, since the binary repulsive interaction between Sn and Ga used for the thermodynamic calculation is not included in the geometrical extrapolation.

It should be noted that interpolation of the liquidus lines in the binary diagrams by straight lines and using this as a basis for extrapolation in the multicomponent regime, as occasionally performed in casting simulations, is an oversimplification and generally not adequate.

For each sample rod, the measured liquidus temperature decreases with higher solute content, Sn leading to a stronger melting point depression than Ga, as already seen in the binary phase diagrams. Most of the DSC slices agree quite well with the liquidus lines (e.g. 1–1, 2–1, 3–1, 4–3, A), others exhibit a deviation of 20 K or more (e.g. 1–2, 1–3, 2–2). To some extent this may be caused by the uncertainty of the EDX measurements, which is estimated to be up to 1 at% in the reported concentration range. For instance, the difference of calculated liquidus temperatures for a Cu_90_Ga_5_Sn_5_ and Cu_88_Ga_6_Sn_6_ alloy is 25 K. Despite these differences in measured and calculated liquidus temperatures, several samples near the same liquidus isotherms exhibit similar liquidus temperatures (e.g. 3–2, 4–2 and 1–1, 2–1, 4–1), showing good agreement with the curvature of the liquidus lines calculated with FactSage and the geometrical extrapolation.

Figure 6 shows an isothermal section of the Cu-rich corner at 1150 K (a) and a solidus projection including the compositions of the DSC samples and the corresponding solidus temperatures calculated by FactSage (b). It is worth noting that a geometrical extrapolation method should not be applied to solidus temperatures, it is potentially grossly erroneous. At 1150 K, three phases appear in the covered concentration range: FCC_A1, BCC_A2 and liquid. Between the respective phase regions, three two-phase regions and one three-phase region exist. The solidus temperatures T_S_ of the Sn-rich samples 1–1 to 2–3, as well as the bulk sample A, are in the range of 1063–1071 K (see Table 3), showing good agreement with the peritectic line at 1072 K in the binary Cu-Sn system^21^. This matches well with the microstructure, since only the two phases α and δ were present in the resolidified samples, but no Cu-Sn γ-phase. For the remaining samples 3–1 to 4–3, T_S_ is increasing with decreasing Sn and increasing Ga concentration, but it is still lower than the solidus temperatures of binary Cu-Ga alloys with comparable Cu content of 80…87 at% which is ≥ 1184 K^24^. Therefore, a small Ga addition to these Cu-Sn alloys only has a minor influence on the peritectic temperature, whereas Sn significantly reduces the peritectic temperature in Ga-rich alloys. This is also confirmed by the calculated solidus projection, as for a Ga content of less than 10 at% the solidus temperatures between the FCC and BCC region are almost independent of the Sn content. Comparing the measured solidus temperatures with the calculated ones, the samples A, 1–1 to 1–3, 2–3 and 3–1 to 4–3 match quite well, while 2–1 and 2–2 deviate strongly. It should be mentioned that interpreting the DSC curves for the measurement of the solidus temperatures is not straightforward, since several signals overlap at temperatures around 1070 K. The highest endothermal signal was selected in the present work. Additional work is needed for a precise determination of the solidus temperatures. However, the potential of the experimental method to assess solidus surfaces is clearly demonstrated.

**Table 3:** Liquidus and solidus temperatures as measured by DSC on sample slices (1–1 to 4–3) and a bulk Cu_87_Ga_2_Sn_11_ sample (A).

Sample	T_L_ (K)	T_S_ (K)
1–1	1237	1068
1–2	1202	1069
1–3	1127	1063
2–1	1243	1069
2–2	1212	1069
2–3	1143	1071
3–1	1259	1102
3–2	1230	1081
3–3	1161	1088
4–1	1246	1197
4–2	1227	1149
4–3	1194	1133
A	1179	1069

Although this technique has proven to be useful, there are some factors limiting its application for more advanced investigations than bulk property mapping and solidus/liquidus surfaces. First, the pulling speed for directional solidification needs to be carefully adjusted, since a Scheil-type profile can only be generated upon plane front solidification. If the speed is set too high, an unstable solidification front will propagate through the sample and cells and dendrites form that change concentration and phase distribution after complete solidification. Second, phases growing during directional solidification will undergo growth competition, and with distance from the starting point a more and more textured microstructure will form, featuring a pronounced anisotropy in all properties. If this is the case, XRD measurements on bulk samples become less reliable for phase identification. This could be circumvented by producing powder samples, for brittle samples e.g. by filing. Third, for investigation of complex equilibria, the rods need to be cut to many slices that can be annealed and quenched to freeze solid state equilibria at different temperatures to create isothermal sections, reducing the benefit of the high-throughput method.

For more complex systems that include numerous intermetallic phases it is reasonable to assume that more DSC samples are needed for investigations of solidus/liquidus surfaces, and the geometrical extrapolation approach will is not expected to be reliable, therefore applying the Calphad method is required. The above listed disadvantages essentially also apply for established high-throughput methods.

Despite those limitations, the technique presented in this work has several advantages, if it is utilized for investigating multicomponent systems since it generates data points that follow paths in the phase diagram. High throughput methods based on vapor deposition and thin films may generate systematic composition variations that can be controlled in a wide range. However, phase formation in thin films can depend on experimental parameters like substrate material, substrate temperature or deposition techniques^36,37^, and metastable states can persist even if post-deposition annealing is performed. In contrast, during mushy-zone resolidification local solid/liquid equilibria are pertained throughout the process, and generally in each isothermal section full thermodynamic equilibrium is adjusted. It has been proven that XRD using the bulk sample material can be sufficient for phase identification even in the presence of textures. This leads to a much faster analysis of microstructures with different compositions, since the entire sample rod can be placed into the XRD device. In the case that phases cannot be identified using the solid rods, the sample has to be sliced to disks which in turn are to be powderized. If texture effects or difficulties in preparation of suitable powders are impeding phase identification with XRD, other techniques like electron backscatter diffraction (EBSD) or diffraction analysis in a transmission electron microscope (TEM) are viable alternatives to determine the phase constitution. In addition to the straightforward determination of bulk hardness values, the cold deformability can be attained by using disks cut out of the rods and a reducing their thickness by a rolling mill.

The four sample rods investigated in the present work yielded over 120 different composition data points for hardness measurements, showing the potential of this technique for high-throughput experimentation. This technique could be utilized in several different fields of application like rapid alloy development, research on multicomponent phase diagrams of unknown or complex systems as well as the improvement of Calphad-type data bases and calculations.

For quaternary and higher component systems, the experimental effort for each sample remains the same, and only a few more samples need to be processed. We expect an increasing advantage of the method with increasing number of alloying elements.

## Conclusions

The high throughput method presented in this paper is capable of covering a wide range of different compositions with limited experimental effort by exploiting extended concentration gradients. It provides the possibility to measure mechanical properties such as hardness as well as thermodynamic data such as solidus and liquidus temperatures in bulk materials. Four samples were sufficient to get the necessary data points for investigating properties of the Cu-rich part of the Cu–Ga–Sn ternary system; the measured Vickers hardness values are in good agreement with the literature values. For the determination of liquidus isotherms, Calphad calculations using FactSage together with a basic ternary database as well as a geometrical extrapolation from the binary systems were carried out and compared with the measured liquidus temperatures of 12 disks cut out of the samples rods. For dilute alloys (in the FCC region), both types of calculated lines agree quite well with each other and the liquidus temperatures of the samples. With increasing solute concentration, the geometrical approach deviates more and more from the FactSage calculation, and the geometrical approach should not be used. For the solidus surface, only the Calphad method can yield meaningful results. In the composition range where intermetallic phases form, extrapolations tend to be more qualitative, independently of the extrapolation method. If ternary phases form, all extrapolations will be erroneous. The experimental method presented in the present work can be applied independently of the complexity of the phase diagram and the interactions of the species.

## Methods

### Materials and experimental set-up

**Figure 7:**
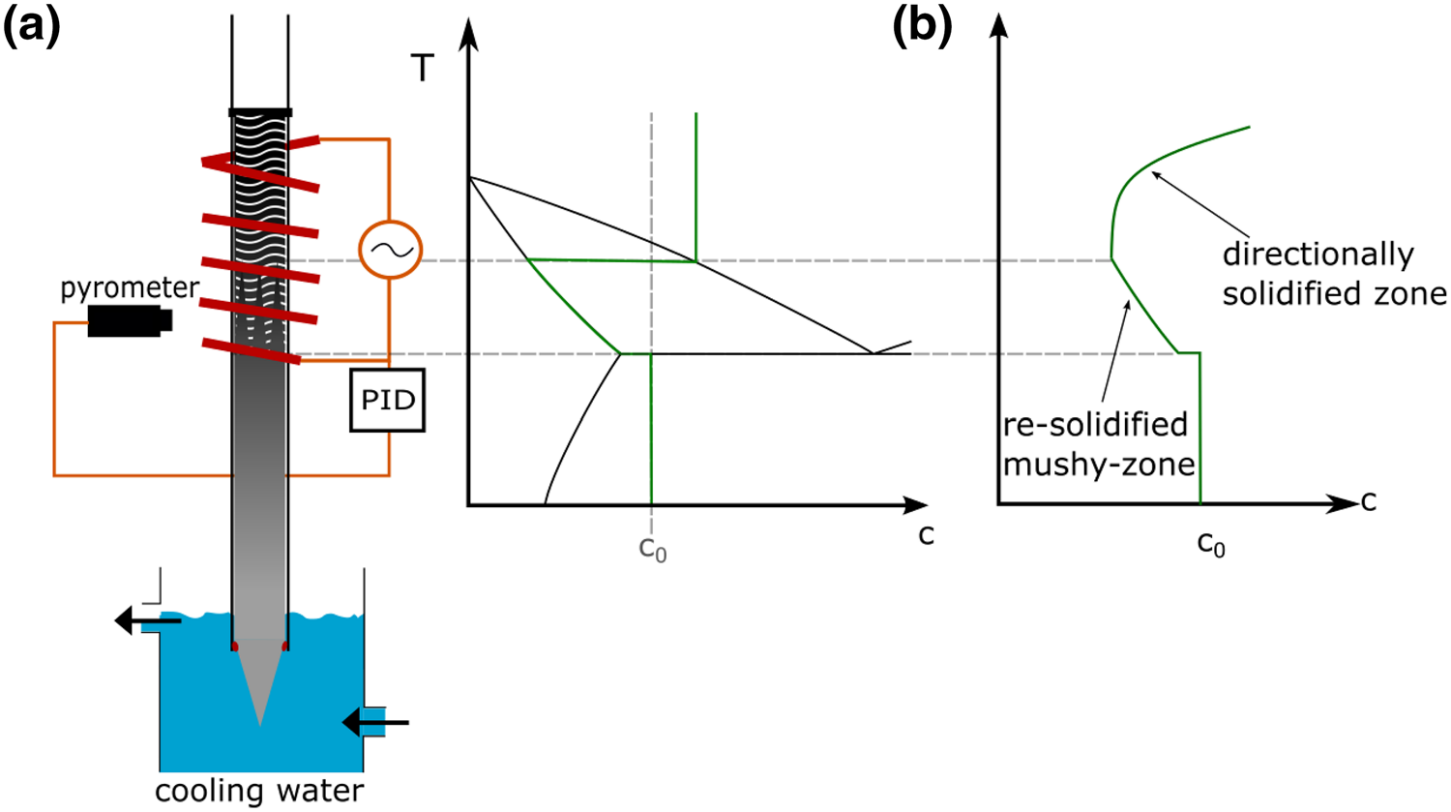
(**a**) Experimental set-up and schematic concentration profiles with respect to the phase diagram in a temperature gradient, and (**b**) concentration profile after mushy-zone resolidification and subsequent directional solidification.

Samples of four different initial concentrations were prepared from the pure elements Cu, Ga and Sn with a purity of 99.99 at%, respectively. Predetermined mixtures of pure elements were melted using induction heating in an alumina crucible. Rods of 8 mm diameter were cast into a preheated mold and cut to 150 mm length; the lower end was shaped to a conical tip for more efficient heat extraction. The rods were inserted into an alumina tube. For the exposure to the temperature gradient, the upper part was placed into a high frequency induction coil and completely melted, while the tip on the lower part was cooled with streaming water, thus remaining solid at all times (Fig. 7a). A pyrometer was used to measure the temperature at 30 mm above the cooling water level, and a constant temperature gradient was maintained by adjusting the heating power of the furnace with a PID controller. The control temperature was set to 800 °C, the water temperature was maintained at 20 °C, leading to a gradient of ~ 26 K/mm. Initially, there is a mushy-zone between the solid part of the sample and the completely liquid zone, with varying solid fraction from f_s_ = 0 at the liquidus temperature and f_s_ = 1 at the solidus temperature. Local equilibration at the solid/liquid interfaces according to the local liquidus and solidus temperatures leads to a concentration gradient, which in turn causes mass transport of solute elements towards higher temperatures^27–31^. The decrease of solute content in the mushy-zone leads to its resolidification, at each location with the local solidus concentration (Fig. 7a), thus forming a macroscopic concentration gradient. The samples were held in the temperature gradient for 6 h to ensure complete mixing laterally, and the complete resolidification of the mushy-zone. Subsequently, a solidification step was carried out by continuously moving the samples into the cooling water with a speed of 1.2 µm/s for 16 h, resulting in a directionally solidified length of 69 mm. The directional solidification occurs under complete mixing in the liquid (due to the inductive heating and the accompanying effective stirring) and negligible diffusion in the solid (due to the extended sample length), resulting in Scheil-type concentration profiles of the solute element concentration (Fig. 7b).

### Processing and analysis

After processing in the temperature gradient, the sample rods were cut longitudinally, ground with SiC paper up to 4000 grit and polished with a suspension of 3 µm diamond particles. A Zeiss Axio Imager light microscope (LM) was used for microstructure analysis and the composition was measured using energy dispersive X-ray spectroscopy (EDX). A Zeiss EVO 40 scanning electron microscope in combination with an EDAX Sapphire Si(Li) detector was used, the measurements were performed with a voltage of 40 kV. For quantification of the EDX spectra, the EDAX Genesis Software with ZAF corrections was used. A Bühler Isomet 500 was used for cutting slices (disks with spherical footprint and a thickness of ~ 1 mm, representing isothermal section) out of the rods, a Bruker D8 Discover with CuKα radiation was utilized for X-ray diffraction measurements for phase determination. Vickers hardness was measured with a Shimadzu HMV-2000 hardness tester, differential scanning calorimetry was carried out using a Mettler Toledo TGA/DSC 1.

## Data Availability

The raw/processed data required to reproduce these findings cannot be shared at this time as the data also forms part of an ongoing study.
